# High hydrostatic pressure induces slow contraction in mouse cardiomyocytes

**DOI:** 10.1016/j.bpj.2022.11.2940

**Published:** 2022-11-28

**Authors:** Yohei Yamaguchi, Masayoshi Nishiyama, Hiroaki Kai, Toshiyuki Kaneko, Keiko Kaihara, Gentaro Iribe, Akira Takai, Keiji Naruse, Masatoshi Morimatsu

## Main text

(Biophysical Journal *121,* 3286–3294; September 6, 2022)

In the original article, reference 70 was missing and should have been added. The reference is as follows:

70. Izumi, M., S. Miyamoto, …, H. Karaki. 2000. Negative inotropic effect of endothelin-1 in the mouse right ventricle. *Eur. J. Pharmacol.* 396:109–117.

In addition, the citation to reference 35 in the third paragraph of the “Morphological changes of cardiomyocytes at high pressure” section on p. 3288 should be replaced with a citation to reference 70. The corrected sentence is as follows: “When focusing on a single twitch elicited by electrical stimulation (i.e., physiological cardiomyocyte contraction), the time to peak twitch of a single twitch, which is the indicator of the physiological contraction speed, is approximately 0.2 s (70).”

