# Supplementary material for: High hydrostatic pressure induces slow contraction in mouse cardiomyocytes
Source: Biophys J. 2022 Jul 14;121(17):3286–94. doi: 10.1016/j.bpj.2022.07.016 (PMC9463647; doi:10.1016/j.bpj.2022.07.016)
Supplement: Document S1. Figures S1–S7 [file mmc1.pdf]

Biophysical Journal, Volume *121*

Supplemental information

High hydrostatic pressure induces slow contraction in mouse cardiomyocytes

Yohei Yamaguchi, Masayoshi Nishiyama, Hiroaki Kai, Toshiyuki Kaneko, Keiko Kaihara, Gentaro Iribe, Akira Takai, Keiji Naruse, and Masatoshi Morimatsu

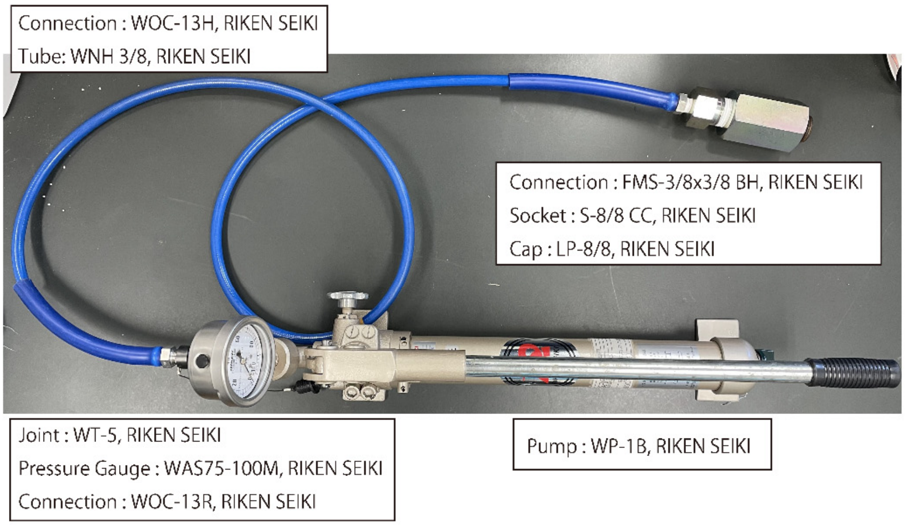

Figure S1. Image of a high-hydrostatic pressure vessel system. The high-hydrostatic pressure vessel system is composed of a pressure vessel and hand pump. The pressure vessel comprises a socket, connection, and cap (S-8/8CC, FMS-3/8×3/8 BH, and LP8/8: RIKEN SEIKI, Japan). The hand pump comprises a pump, pressure gauge, joint, and connection (WP-1B, WAS75-100M, WT-5, and WOC-13R: RIKEN SEIKI). The pressure vessel and the hand pump are connected by a tube and a connection (WNH 3/8 and WOC-13H: RIKEN SEIKI). The cells isolated from the mouse heart are placed in the pressure vessel, and the hand pump is used to apply pressure to the vessel. High hydrostatic pressure compresses the cardiomyocytes in the pressure vessel during this application, as described in the Materials and Methods section.

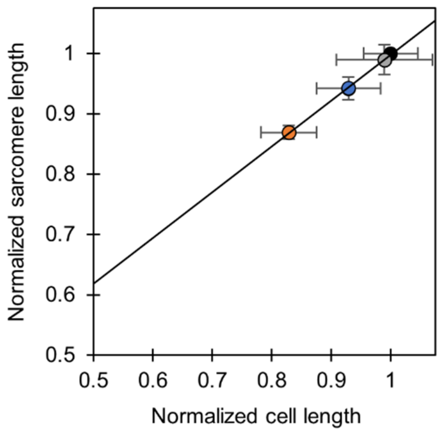

Figure S2. Correlation between cell length and sarcomere length at 0.1 (black; n = 12), 5 (gray; n = 5), 10 (blue; n = 8), and 20 MPa (orange; n = 14). Cell length is highly correlated to SL (R^2 = 0.99). The cell length and SL are normalized to the initial value (t = 0) at pressures. Each datapoint represents the mean and the error bars represent the SEM.

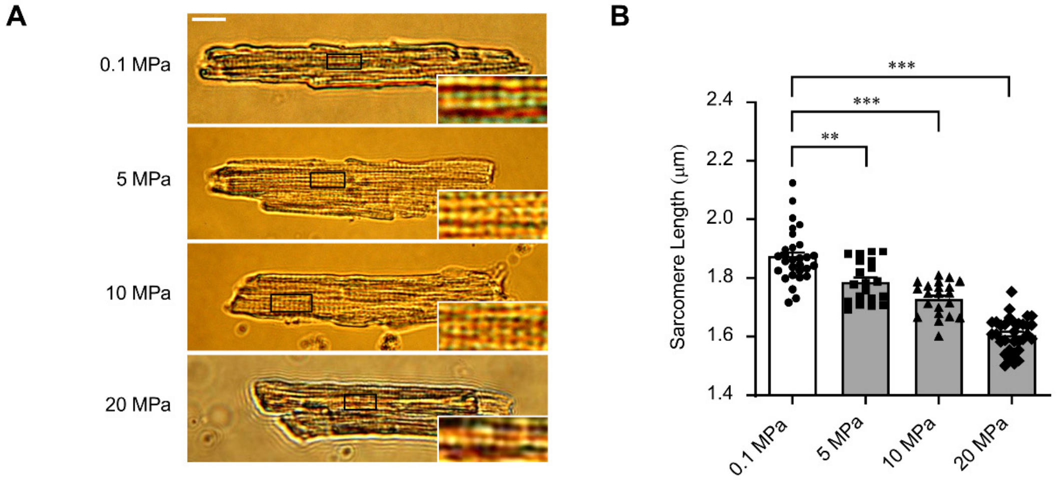

Figure S3. Morphological change in cardiomyocytes under high hydrostatic pressure using chemical fixation (A) Images of cardiomyocytes chemically fixed after exposure to atmospheric pressure (0.1 MPa) and high pressures (5, 10, and 20 MPa). The black inset is magnified in the lower right corner of the image. Scale bar: 20 µm. (B) Dose-dependent shortening of SL under high-pressure conditions (5, 10, and 20 MPa). Myocytes were

exposed to 0.1 MPa (n = 29 cells), 5 MPa (n = 20 cells), 10 MPa (n = 21 cells), and 20 MPa (n = 32 cells) pressure for 5 min. Significant shortening was observed at high-pressure conditions (5, 10, and 20 MPa). ** $p < 0.01$, *** $p < 0.001$.

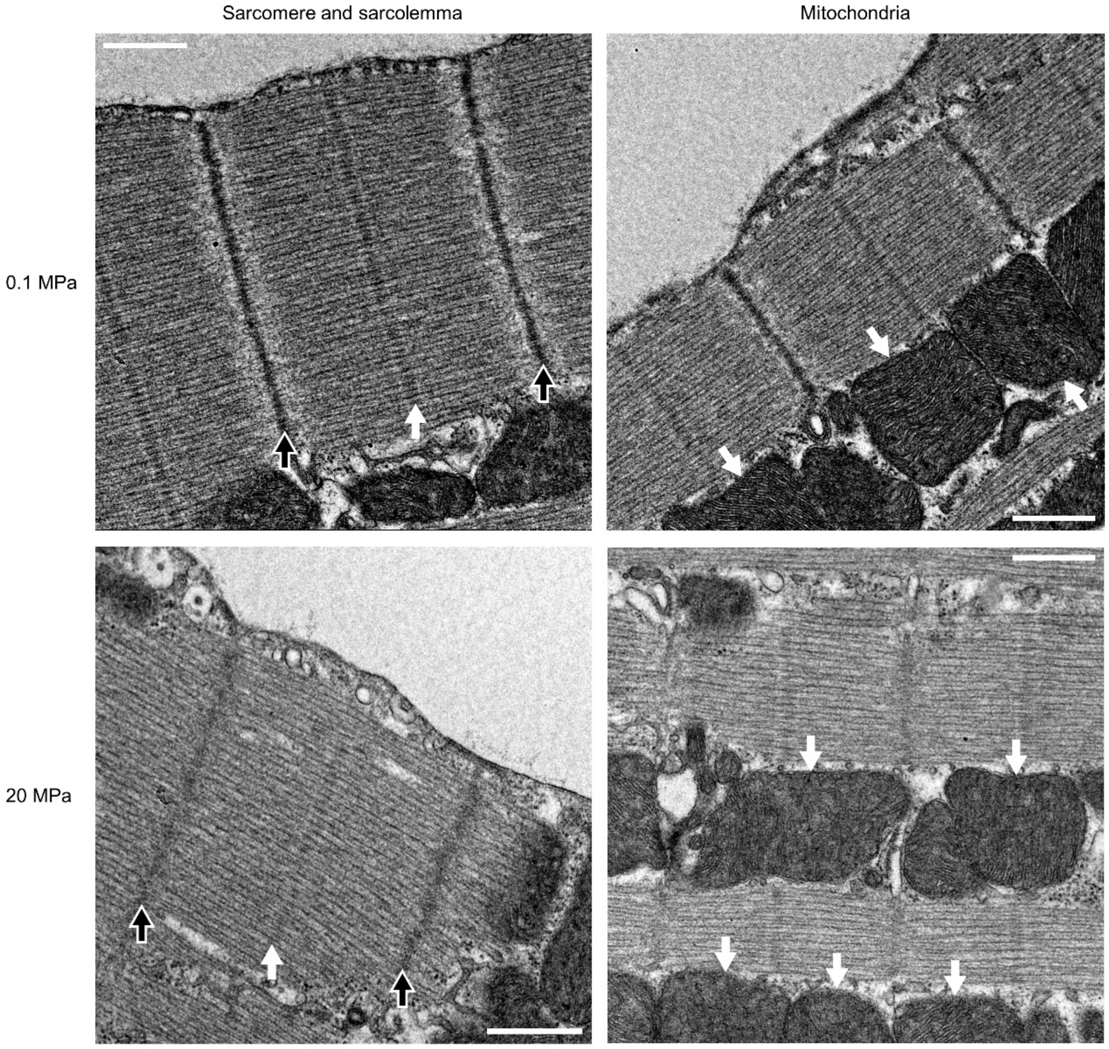

Figure S4. Transmission electron microscope (TEM) images of sarcomere, sarcolemma, and mitochondria structures in cardiomyocytes. TEM images of single cardiomyocytes were chemically fixed after exposure to atmospheric pressure (0.1 MPa) or high pressures (20 MPa). The sarcolemma and sarcomere structures, including the M line (white arrows)

and Z line (black arrows), and the inner structure of the mitochondria (white arrows) are clearly observed at atmospheric pressure (upper panels). At a pressure of 20 MPa, sarcolemma and sarcomere structures are not collapsed, while the inner structure of mitochondria (cristae) is slightly disrupted (lower panels). Scale bar: 500 nm.

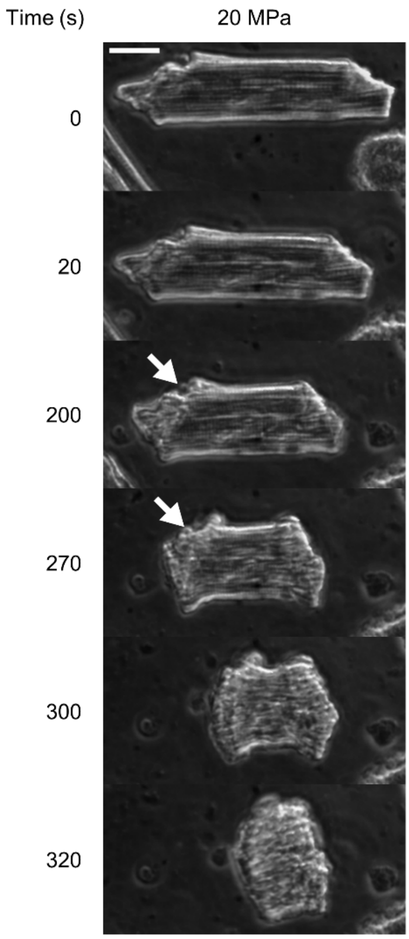

Figure S5. The collapse of cardiomyocytes under high pressure at 20 MPa. Sequential images of cardiomyocyte morphological changes under high pressure at 20 MPa show the pressure-induced cell collapse during the application from 200 to 320 s. White arrows indicate small blebbing, during which the cardiomyocytes lose their highly organized three-dimensional structure, round up, and collapse. Scale bars: 20 μm. Refer to Movie S5.

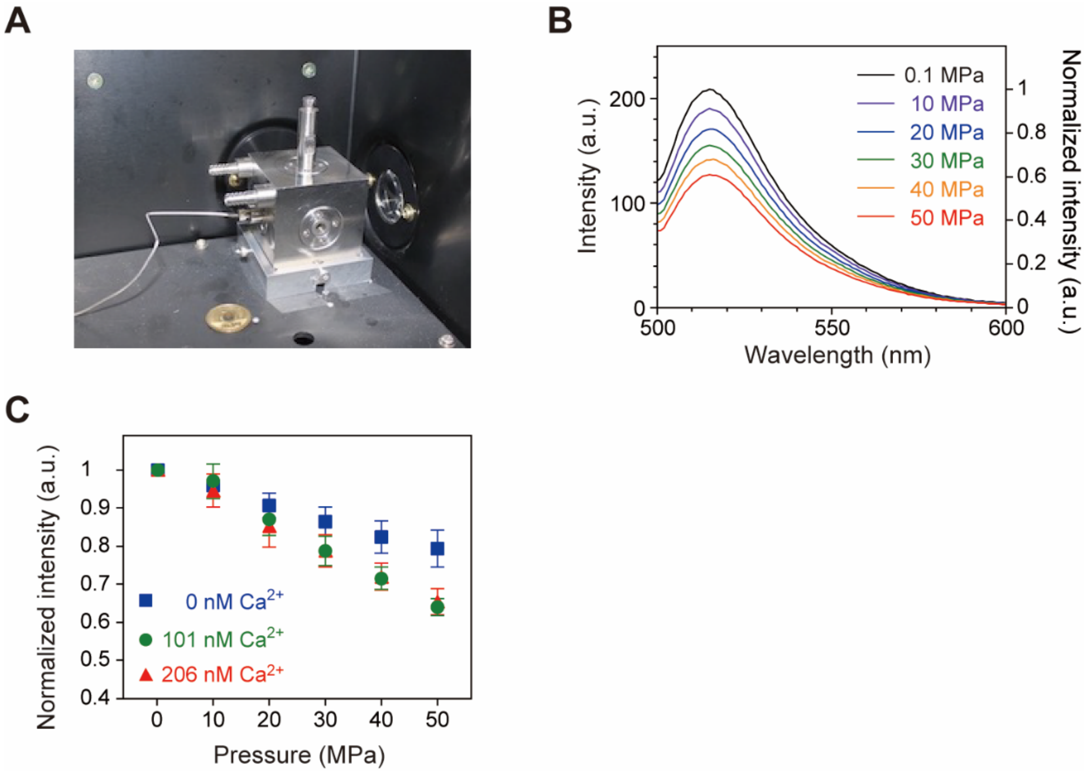

Figure S6. Effect of hydrostatic pressure on fluorescence indicator of Cal-520. (A) High-pressure chamber for spectroscopy (the diameter of the coin = 22 mm). The chamber was combined with a fluorescence spectrophotometer (F-2500, Hitachi, Japan). (B) Emission spectra of Cal-520 measured at pressures ranging from 0.1 to 50 MPa in 101 nM free Ca^{2+} concentration. The fluorescence intensities were normalized to peak values at 0.1 MPa. (C) Pressure dependence of the peak intensities at each free Ca^{2+} concentration in the buffer solution at 0.1 MPa (n = 4). Error bars represent the standard deviation.

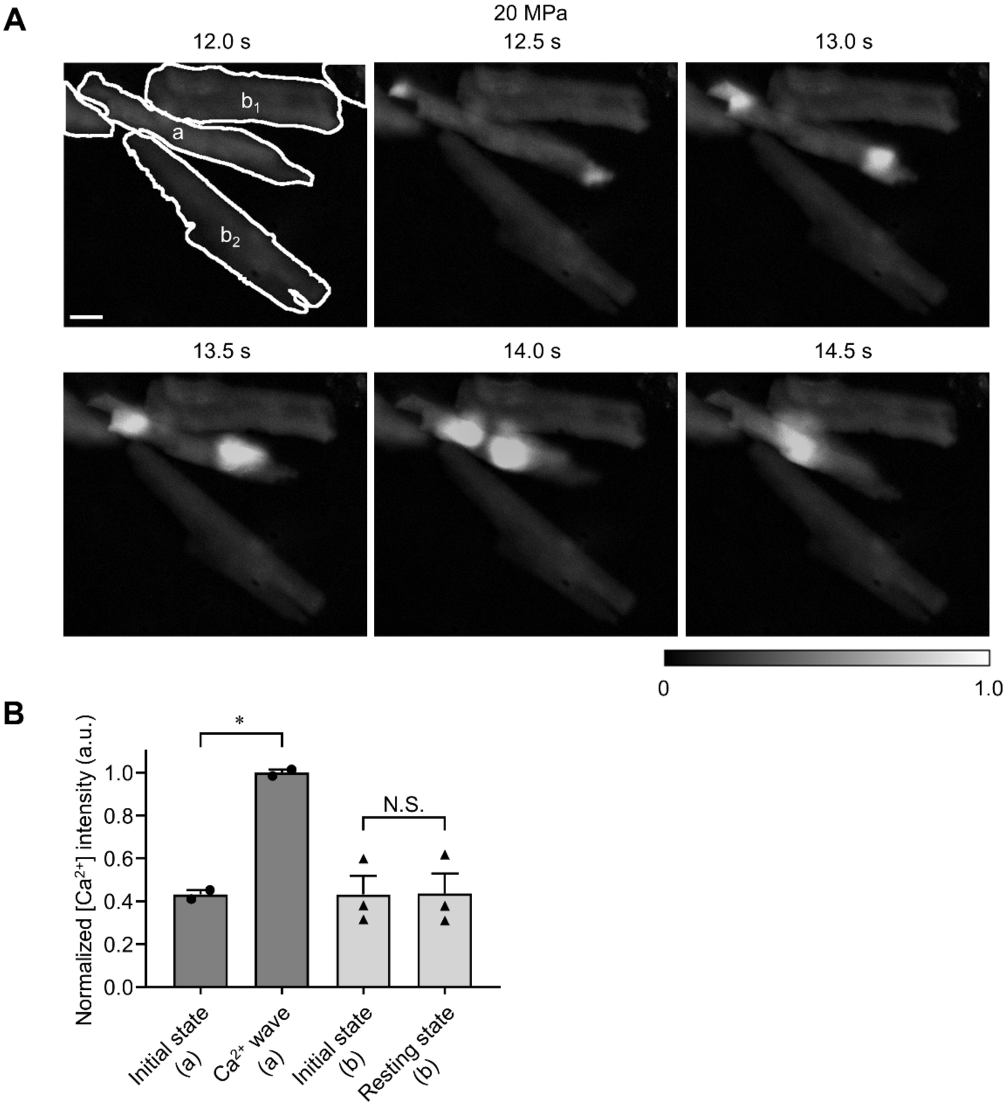

Figure S7. Qualification of detecting the acute change in cytosolic Ca^{2+} using Cal-520 AM. (A) Sequential grayscale images of cytosolic Ca^{2+} change in the cells represent Ca^{2+} wave (a) or the cells at rest (b_1 and b_2). The alteration of [Ca^{2+}] intensity is represented in grayscale. Scale bars: 20 μm. (B) In the cells with Ca^{2+} wave (a), when the Ca^{2+} wave appeared, the [Ca^{2+}] intensity was significantly elevated compared with that at an initial state. In the cells at rest (b), no significant increase in [Ca^{2+}] intensity was observed. * $p < 0.01$ vs initial state. N.S.: not significant. Refer to Movie S7.
