# Supplementary material for: High hydrostatic pressure induces slow contraction in mouse cardiomyocytes
Source: Biophys J. 2022 Jul 14;121(17):3286–94. doi: 10.1016/j.bpj.2022.07.016 (PMC9463647; doi:10.1016/j.bpj.2022.07.016)
Supplement: Document S2. Article plus supporting material [file mmc11.pdf]

Biophysical Journal
Article

High hydrostatic pressure induces slow contraction in mouse cardiomyocytes

Yohei Yamaguchi,[1,2,*] Masayoshi Nishiyama,[3] Hiroaki Kai,[1] Toshiyuki Kaneko,[2] Keiko Kaihara,[1] Gentaro Iribe,[1,2] Akira Takai,[2] Keiji Naruse,[1] and Masatoshi Morimatsu[1,*]
[1]Department of Cardiovascular Physiology, Graduate School of Medicine, Dentistry and Pharmaceutical Sciences, Okayama University, Okayama, Japan; [2]Department of Physiology, Asahikawa Medical University, Asahikawa, Hokkaido, Japan; and [3]Department of Physics, Faculty of Science and Engineering, Kindai University, Higashiosaka, Osaka, Japan

ABSTRACT Cardiomyocytes are contractile cells that regulate heart contraction. Ca^{2+} flux via Ca^{2+} channels activates actomyosin interactions, leading to cardiomyocyte contraction, which is modulated by physical factors (e.g., stretch, shear stress, and hydrostatic pressure). We evaluated the mechanism triggering slow contractions using a high-pressure microscope to characterize changes in cell morphology and intracellular Ca^{2+} concentration ($[Ca^{2+}]_i$) in mouse cardiomyocytes exposed to high hydrostatic pressures. We found that cardiomyocytes contracted slowly without an acute transient increase in $[Ca^{2+}]_i$, while a myosin ATPase inhibitor interrupted pressure-induced slow contractions. Furthermore, transmission electron microscopy showed that, although the sarcomere length was shortened upon the application of 20 MPa, this pressure did not collapse cellular structures such as the sarcolemma and sarcomeres. Our results suggest that pressure-induced slow contractions in cardiomyocytes are driven by the activation of actomyosin interactions without an acute transient increase in $[Ca^{2+}]_i$.

SIGNIFICANCE Cardiomyocytes are contractile cells in the heart. Physiological contraction is regulated by activation of actomyosin interaction by an acute transient increase in $[Ca^{2+}]_i$. This behavior is expected to be modified by high hydrostatic pressure. In this study, we have reported that high pressure induces slow contraction in mouse cardiomyocytes. We revealed that the pressure-induced slow contraction was initiated by the activation of actomyosin interactions without an acute transient increase in $[Ca^{2+}]_i$. The present study is the first report on cardiomyocyte behavior at high-pressure levels.

INTRODUCTION

Cardiomyocytes are contractile cells that regulate contraction in the heart. Physiological cardiomyocyte contraction is prohibited in the absence of intracellular Ca^{2+} since myosin-binding sites on actin filaments are tightly covered by the tropomyosin-troponin complex (1–6). Therefore, the myosin heads of the filaments are unable to interact with actin filaments. Ca^{2+} enters the cell through activated Ca^{2+} channels, which subsequently initiates Ca^{2+} release from the sarcoplasmic reticulum, the process of calcium-induced calcium release (CICR). CICR leads to an acute transient increase in the intracellular Ca^{2+} concentration ($[Ca^{2+}]_i$), which allows Ca^{2+} to bind to troponin C, triggering conformational changes of the tropomyosin-troponin complex and leading to actomyosin interactions (1–9). This behavior induces cardiac contraction, which is modulated by several physical conditions, such as stretch, shear stress, and temperature (10–16). In addition to these physical factors, the hydrostatic pressure of 15–25 kPa (i.e., blood pressure) is considered to be an essential modulator of cardiac function (17–20). Thus, high hydrostatic pressure at the megapascal level is also predicted to modify cardiac contraction. This idea is well supported by previous experiments on intact tissues (21–26) and purified proteins (27–29) derived from striated muscle having sarcomere structures. Hydrostatic pressure at tens of megapascals could trigger muscle contraction at the tissue level by promoting tropomyosin to shift its position on the actin

Submitted January 17, 2022, and accepted for publication July 11, 2022.
*Correspondence: yyamaguchi@asahikawa-med.ac.jp or mmorimatsu@okayama-u.ac.jp
Yohei Yamaguchi and Masatoshi Morimatsu contributed equally to this work.
Editor: Jeffrey Saucerman.
https://doi.org/10.1016/j.bpj.2022.07.016
© 2022 Biophysical Society.
This is an open access article under the CC BY license (http://creativecommons.org/licenses/by/4.0/).

filament. However, cellular dynamics under high-pressure conditions are still unclear due to the lack of a quantitative measurement device.

Here, we investigated changes in the cellular dynamics of live cardiomyocytes under high hydrostatic pressure using a high-pressure microscope. Our study reports that high pressure causes cardiomyocyte contraction by promoting actomyosin interaction without an acute transient increase in $[Ca^{2+}]_i$ derived from CICR.

MATERIALS AND METHODS

Myocyte isolation

All experimental procedures were conducted in accordance with the Guiding Principles for the Care and Use of Animals approved by the Council of the Physiological Society of Japan. The Animal Subjects Committee of Okayama University Graduate School of Medicine, Dentistry, and Pharmaceutical Sciences approved the animal experimental procedures. Ventricular cardiomyocytes were isolated from the hearts of 18 male mice (C57BL/6J) aged 8–12 weeks, as described previously (30). In brief, adult mice were anesthetized with isoflurane (DS Pharma Animal Health, Osaka, Japan) and injected intraperitoneally with 100 IU of heparin sodium (AY Pharmaceuticals, Tokyo, Japan). The mice were euthanized using isoflurane at 30 min postinjection. The heart was immediately excised and perfused on a handmade Langendorff apparatus with oxygenated solution A (128 mM NaCl, 2.6 mM KCl, 1.18 mM $MgSO_4$, 1.18 mM KH_2PO_4, 10 mM HEPES, 20 mM taurine, and 11 mM glucose [pH 7.4], adjusted with NaOH) containing 10 mM 2,3-butanedione monoxime (BDM) (Sigma-Aldrich, St. Louis, MO) at 36–38°C. Ventricular cardiomyocytes were isolated using the enzyme Liberase TM Research Grade (Roche, Basel, Switzerland). The isolated cells were placed in a storage solution (solution A containing 1.8 mM $CaCl_2$) for at least 30 min before the measurement.

High-pressure microscopy

We developed a high-pressure microscope optimized for achieving the best images and stabilizing samples under high pressure up to 150 MPa, as reported previously (31). This microscope allowed us to visualize the effects of hydrostatic pressure on research targets, including molecular machines, cells, and organisms (32–35). The high-pressure chamber for optical microscopy was connected to a hand pump. The pressure apparatus was combined with an inverted microscope (IX70; Olympus, Tokyo, Japan). Microscopic observations were carried out using a long working distance objective lens (LUCPlanFLN 40×; Olympus). Bright-field images were recorded with an EMCCD camera (Andor iXon X3; Oxford Instrument, Abingdon, UK) at 1 frame s^{-1} and analyzed offline using Fiji/ImageJ software (https://imagej.net/Fiji).

Isolated myocytes were put in normal Tyrode solution (140 mM NaCl, 5.4 mM KCl, 1.8 mM $CaCl_2$, 1.0 mM $MgCl_2$, 5.0 mM HEPES, and 11 mM glucose [pH 7.4], adjusted with NaOH) in the high-pressure chamber. All experiments were conducted at room temperature (23–25°C). The cardiomyocyte morphology and sarcomere length (SL) were observed at pressures of 5, 10, or 20 MPa. The pressure was controlled with an accuracy of ± 1 MPa. The total elapsed time for pressure treatment of a cell population was 3 min. After the release of the pressure, all cells were removed from the chamber and the assay was repeated using the cells that had not been exposed to high pressure.

The combination of the high-pressure microscope and measurement of the SL, which is a suitable indicator of cell length, has allowed us to observe accurate cellular kinetics in live cardiomyocytes under high pressure (36,37). SL was analyzed offline using a SarcOptiM plug-in for Fiji/ImageJ software (http://pccv.univ-tours.fr/ImageJ/SarcOptiM) according to the provided instructions (36). The plug-in was developed to easily calculate the SL from live-cell images using an optical microscope instead of measuring the cell length, on which there is great variability among cardiomyocytes. From the images, a region of interest was selected along the long axis of the cell. The fast Fourier transform (FFT) spectrum of the sarcomere was calculated from the profile along the longitudinal axis of the myocytes. The corresponding SL was then determined by considering the X coordinate of the peak in the FFT spectrum and the pixel size (0.4 μm).

High-hydrostatic pressure vessel system

A high-hydrostatic pressure vessel system (see Fig. S1) was used to apply high pressure to cells. The system was composed of a pressure vessel and a hand pump (S-8/8CC, WP-1B; RIKEN SEIKI, Japan). Isolated cardiomyocytes were inserted into the pressure vessel, and the vessel cap (LP-8/8; RIKEN SEIKI) was tightly closed. Then, hydrostatic pressure was applied using the hand pump. The hydrostatic pressure in the vessel could be increased and decreased in increments of 5 MPa. High-hydrostatic pressure was maintained for 5 min. Immediately after releasing the pressure, the cells were fixed with 4% paraformaldehyde in phosphate-buffered saline (PBS) overnight at room temperature (23–25°C). After cell fixation, the SL was measured using IonOptix hardware and software (IonOptix Corporation, Milton, MA) and an inverted microscope (IX70; Olympus), as described previously (30).

Transmission electron microscopy

The effect of high hydrostatic pressure on intracellular structures was investigated using transmission electron microscopy (TEM). The specimens were prepared for TEM as described previously (38). In brief, ambient pressure (0.1 MPa) or high hydrostatic pressure (20 MPa) was applied to the isolated cardiomyocytes for 5 min using the high-hydrostatic pressure vessel system. Immediately after releasing the high pressure, the isolated ventricular myocytes were placed in half Karnovsky solution (2.5% glutaraldehyde and 2% paraformaldehyde in PBS [pH 7.4]) and fixed overnight at room temperature (23–25°C). The fixed cells were embedded in a low-viscosity acrylic embedding medium (LR white resin; Agar Scientific, Stansted, UK). The resin block was sectioned to a thickness of 90 nm. The tissue contrast was enhanced by exposure to 2% aqueous uranyl acetate for 5 min, after which it was rinsed in ultrapure sterile water and exposed for 5 min to lead citrate. The sections were imaged using a transmission electron microscope (H-7650; Hitachi, Tokyo, Japan).

High-pressure fluorescence spectroscopy

A high-pressure optical chamber was constructed for absorption and fluorescence spectroscopy measurements as reported previously (39,40). The chamber was connected to a hand pump (HP-150; Syn Corporation, Kyoto, Japan). The chamber was then placed inside a fluorescence spectrometer (F-2500; Hitachi). The sample solution was enclosed in an inner cuvette (optical pathlength, 4 mm; inner volume, ~0.25 mL) and inserted into the main body of the chamber. The excitation wavelength used was 488 nm. The emission spectra were scanned from 500 to 600 nm for a Ca^{2+} indicator (Cal-520 sodium salt; AAT Bioquest, Sunnyvale, CA). The indicator concentration was 5 nM in EGTA-buffered solutions (30 mM HEPES, 6.2 mM $MgCl_2$, and 30 mM EGTA) containing different concentrations of free Ca^{2+} (0, 101, and 206 nM prepared by adding 0, 10, and 14.5 mM $CaCO_3$, respectively) at 0.1 MPa. The pH of each solution was adjusted to 7.2 using KOH. All the experiments were performed at room temperature (23–25°C). The indicator concentration was corrected based on the density of the distilled water at each pressure point (41).

Calcium imaging

Cal-520 acetoxymethyl ester (Cal-520 AM; AAT Bioquest) was used as an organic Ca^{2+} dye to record $[Ca^{2+}]_i$, as described previously (42). Isolated cardiomyocytes were dye-loaded at room temperature (23–25°C) by incubation for 30 min with 4.5 μM Cal-520 AM in normal Tyrode solution, and then the cells were washed with normal Tyrode solution for 30 min before imaging. The cytosolic Ca^{2+} signal was measured on the abovementioned high-pressure microscope. Cells were exposed to ambient pressure (0.1 MPa) or high pressure (20 MPa) in normal Tyrode solution. Cal-520 was excited at a wavelength of 470–495 nm using a mercury lamp (Filter cube: U-FBNA; Olympus). Light emitted at a wavelength of 510–550 nm was captured using the EMCCD camera. Images were acquired at 10 frame s^{-1}. After collecting the images, the signal intensity was measured offline using the Fiji/Image J software.

Statistical analysis

The results are expressed as mean ± standard error of the mean (SEM). All results were compared using Student's paired *t*-test or one-way analysis of variance with Bonferroni's or Dunnett's post hoc test, as appropriate. Statistical significance is defined for each experiment, with default set to $p < 0.05$.

RESULTS AND DISCUSSION

Morphological changes of cardiomyocytes at high pressure

Live ventricular cardiomyocytes, enzymatically isolated from the hearts of C57BL/6J mice, were visualized under high-pressure conditions using a high-pressure microscope. The cardiomyocytes were introduced into a high-pressure chamber for optical microscopy and the pressure-induced changes of cell shape and SL were observed. At 0.1 MPa, the cell length and width (the cell long and short axis, respectively) remained constant with time (Video S1). At 5–20 MPa, the cell length slowly decreased by ~17% and the cell width increased by ~11% relative to the low-pressure controls, indicating that high pressure induces slow contraction compared with the physiological contraction (Fig. 1 *A*, Videos S2–S4, and Table 1). In physiological myocyte contraction, the cell length decreases within 1 s by ~12% while the cell width increases by ~6% relative to the relaxed cells as the total volume of the intact muscle remains constant (43–45), indicating that pressure-induced cell shortening (i.e., change in cell morphology) resembles physiological contraction with electrical stimulation derived from cardiac pacemaker cells.

The SL was measured to provide a more accurate indicator of cell contraction since the SL is representative of cell length. The position of the peak in the FFT spectrum of the sarcomere was shifted (Fig. 1 *A*), showing a decrease in the SL when the cell length shortened under high pressure. Data of SL versus cell length were fitted to a line with a slope of 0.79 ± 0.09 (mean ± fitting error), demonstrating that the change in SL was highly correlated with the change in cell length (Fig. S2).

Fig. 1 *B* displays time courses of SL at each pressure. At 0.1 MPa, the SL remained constant with time. On the other hand, the SL slowly decreased with time at 5–20 MPa. When applying pressure of 5 MPa, the SL slightly shortened with time, while a significant decrease in SL was not detected at its end state. The SL significantly decreased with increasing pressure at the end state under pressure of 10 and 20 MPa (Fig. 1 *C*). In addition, the high pressure decreased the time constant calculated from the exponential curve fitting, which is an indicator of the monophasic cell contraction speed, from 70 s at 5 MPa to 5 s at 20 MPa (Fig. 1 *B*), indicating that the pressure-induced contraction speed accelerated with an increase in pressure from 5 to 20 MPa. When focusing on a single twitch elicited by electrical stimulation (i.e., physiological cardiomyocyte contraction), the time to peak twitch of a single twitch, which is the indicator of the physiological contraction speed, is approximately 0.2 s (35). Therefore, the cell contraction speed was entirely different between the pressure-induced slow contraction and physiological contraction, although the percentage changes in cell length and width in the pressure-induced slow contraction were similar to those in the physiological contraction, as described above, suggesting that pressure-induced slow contraction resulted from a mechanism different from that of physiological contraction.

Pressure-induced contraction was also confirmed using a high-pressure vessel system. The cardiomyocytes were introduced into the high-hydrostatic pressure vessel system. The measured SL was 1.88 ± 0.02 μm (mean ± SEM, $n = 29$) under ambient conditions (Fig. S3), which is consistent with previously reported values of SL (42,46) and confirms that cell shortening is not observed in the absence of pressure. Hydrostatic pressures of 5, 10, and 20 MPa were applied for 5 min to the cells in the pressure vessel. Immediately after releasing the pressure, the cells were chemically fixed with 4% paraformaldehyde in PBS. Subsequently, the detailed cellular structure and SL were monitored using an optical microscope. Clear sarcomere structure, forming a striped pattern, was observed in these cardiomyocytes (Fig. S3 *A*). The pressure treatment at 20 MPa for 5 min shortened the SL (1.61 ± 0.01 μm [mean ± SEM, $n = 32$]) compared with the slack SL values. The SL monotonically decreased with increasing pressure (Fig. S3 *B*).

We further examined the alteration of the sarcomere structure under high pressure at high resolution using TEM. TEM images of cells exposed to 20 MPa show that sarcomere structures, the region between two Z lines, were shortened, and light isotropic (I) bands were not detected (Fig. 1 *D*). At the same time, the sarcomere and sarcolemma did not collapse. In contrast, images collected at 0.1 MPa show a central dark anisotropic (A) band and two adjacent half I bands in the sarcomere, indicating

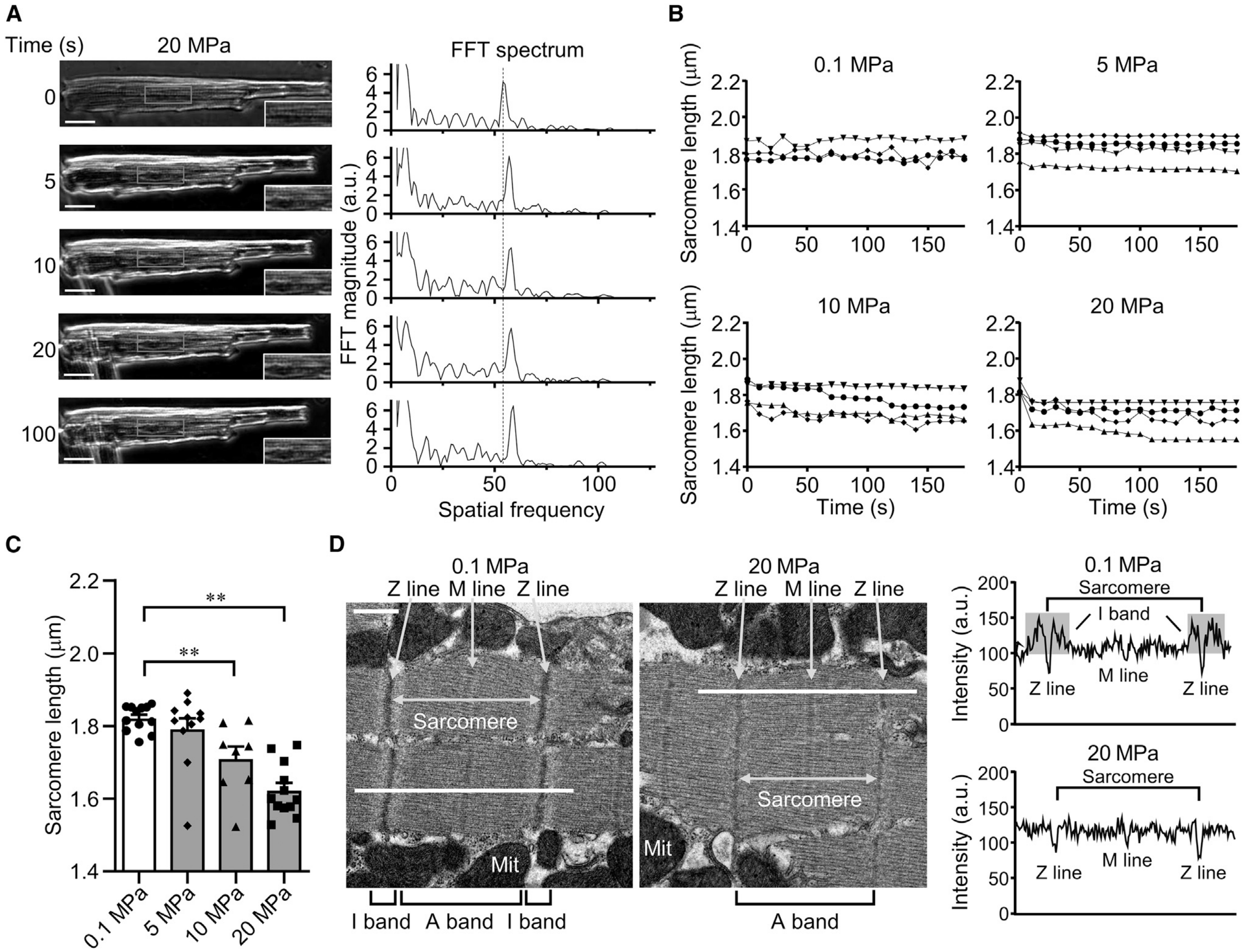

FIGURE 1 High hydrostatic pressure induced slow contraction in mouse cardiomyocytes. (*A*) Cardiomyocyte images and their computed FFT spectrum of sarcomere during the application of the pressure at 20 MPa. The gradual shift of the peak position in the FFT spectrum to higher frequency corresponds to a decrease in the SL. The black inset is magnified in the lower right corner of the image. Scale bar, 20 μm. (*B*) The time-dependent SL alteration under ambient pressure (0.1 MPa) and high pressure (5, 10, and 20 MPa). The SL gradually declined under pressures of 5, 10, and 20 MPa, while it remained constant under ambient pressure. Each different data point represents the different cells. (*C*) Comparison of SL under each pressure condition (5, 10, and 20 MPa). Pressures of 5, 10, and 20 MPa ($n = 11$, 8, and 12 cells, respectively) led to a decrease in SL compared with the ambient pressure of 0.1 MPa ($n = 12$ cells). $^{**}p < 0.01$. Images recorded at 1 frame s^{-1}. Refer to Videos S1–S4. (*D*) TEM images of a single cardiomyocyte before and after the application of 20 MPa pressure. The images show that the I band was clear at a pressure of 0.1 MPa, whereas it was undetectable at a pressure of 20 MPa. No collapse of sarcomere structures (A band and Z line) was observed. Quantification of intensities derived from the white line on each image. The lowest intensity peak (*darker line*) corresponds to the Z line, and the second-lowest intensity peak (*lighter line*) corresponds to the M line. Although the I band was detected at 0.1 MPa pressure, it disappeared after the application of high pressure at 20 MPa, indicating cardiomyocyte contraction. Scale bar, 500 nm.

the slack SL of the cardiomyocytes (Figs. 1 *D* and S4). No remarkable changes have been observed in the structure of the sarcomere and sarcolemma in striated muscle with pressures of up to 100 MPa (47). These findings are consistent with the results we obtained at 20 MPa, suggesting that high pressure induces cardiomyocyte contractions without any collapse in sarcomere and sarcolemma structures.

We observed that the application of pressures at 20 MPa disrupted cardiomyocyte structure in several experiments (Fig. S5 and Video S5). These cardiomyocytes with membrane damage by extremely high collagenase and protease

TABLE 1 Normalized cell length and width using the initial value (t = 0 s) at pressures

	0.1 MPa ($n = 13$)	5 MPa ($n = 5$)	10 MPa ($n = 8$)	20 MPa ($n = 19$)
Cell length	1.00 ± 0.05	0.99 ± 0.07	0.93 ± 0.05	0.83 ± 0.04
Cell width	1.00 ± 0.07	1.05 ± 0.03	1.07 ± 0.09	1.11 ± 0.06

Values are mean ± SEM.

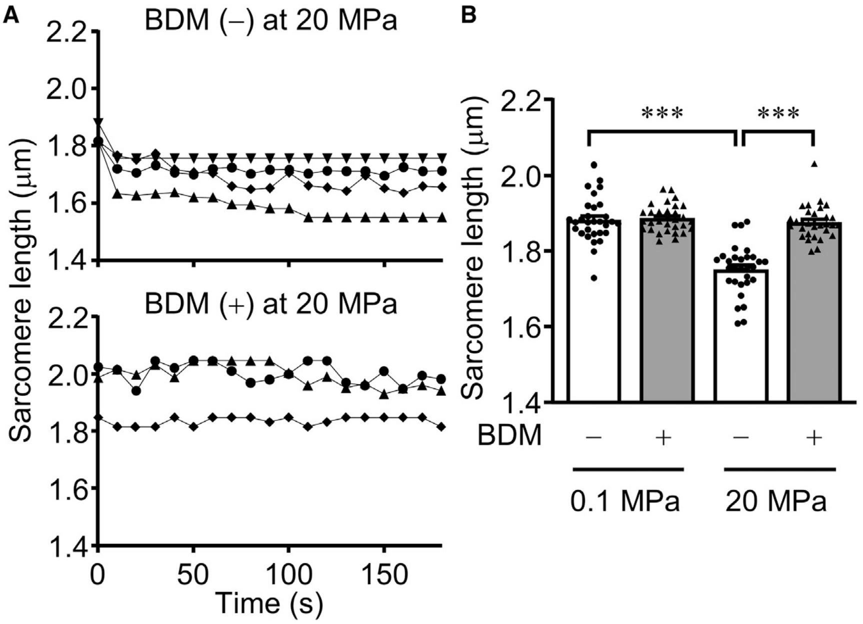

FIGURE 2 Effects of myosin ATPase inhibitor (BDM) on the pressure-induced slow contraction. (*A*) The pressure-induced slow contraction in live cardiomyocytes is observed in normal Tyrode solution during the application of pressure (*upper panel*), while its slow contraction is not observed in BDM-containing Tyrode solution (*lower panel*). Images were recorded at 1 frame s^{-1}. Refer to Videos S4 and S6. (*B*) BDM significantly inhibits the pressure-induced shortening in cells chemically fixed after the pressure treatment ($n = 30$ cells), while the shortening is still observed in the absence of BDM ($n = 30$ cells). ***$p < 0.001$.

activities during cell isolation may be caused by the slow contraction at high pressure (48). Pressure application experiments were performed on cells with no observed structural disruption. Thus, cellular function in the cardiomyocytes would be preserved under high pressure when pressure-induced slow contraction is observed in the cardiomyocytes.

Pressure-induced slow contraction inhibited by myosin ATPase inhibitor

The molecular mechanism of actomyosin interaction in cardiomyocyte contraction is well studied (49,50). At high concentrations (50 mM), the non-selective myosin ATPase inhibitor BDM weakens the interaction between actin and myosin by stabilizing the myosin-ADP-Pi intermediate (51–53). We investigated the effects of BDM on live cardiomyocytes at 20 MPa. High-pressure microscopy revealed the time course of SL changes in the BDM-containing Tyrode solution (Fig. 2 *A* and Video S6). Although the SL gradually decreased with time in the absence of BDM (upper panel in Fig. 2 *A*), applied pressure of 20 MPa did not significantly change the SL in the presence of BDM (lower panel in Fig. 2 *A*).

Next, we further verified the effects of BDM on cells chemically fixed following pressure treatment. Pressure of 20 MPa significantly decreased the SL on the cells in the absence of BDM, whereas the pressure-induced shortening of the SL was significantly suppressed with 50 mM BDM in Tyrode solution, as observed in live cells (Fig. 2 *B*). Cardiomyocytes, therefore, contracted at high pressure with slow speed, which was generated by the actomyosin interaction activated by high pressure.

Calcium imaging of cardiomyocytes exposed to high pressure

Cardiomyocyte contraction occurs as a result of actomyosin interaction alterations, which are regulated by acute transient changes in $[Ca^{2+}]_i$ (7–9). We investigated the relationship between pressure-induced slow contractions and an acute transient increase in cytosolic Ca^{2+} levels in cardiomyocytes. The change in the cytosolic Ca^{2+} at high-pressure levels was assessed by measuring the Cal-520 fluorescence intensity in cardiomyocytes with high-pressure microscopy. We first confirmed the effect of high hydrostatic pressure on the Cal-520 fluorescence intensity. The emission spectra were recorded at pressures ranging from 0.1 to 50 MPa. High pressure tends to decrease the fluorescence intensity, which is similar to previously reported results using the different fluorescence indicators of Fluo-4 (54). The pressure of 20 MPa reduced the intensity of Cal-520 by only 13–15% when the free Ca^{2+} concentration was 100–200 nM, which assumes a diastolic internal Ca^{2+} concentration of 150 nM (Fig. S6) (7). Furthermore, we confirmed the acute change in cytosolic Ca^{2+} fluorescence intensity during the Ca^{2+} wave using the high-pressure microscope (see Fig. S7 and Video S7). Subsequently, cytosolic Ca^{2+} fluorescence intensities were recorded under each pressure condition (0.1 and 20 MPa). Fig. 3 *A* shows sequential grayscale Ca^{2+} images of the same cardiomyocyte at 20 MPa. Although the cells contracted slowly at 20 MPa, the cytosolic Ca^{2+} fluorescence intensity showed no acute transient change (Fig. 3 *B* and Video S8). Similarly, the cytosolic Ca^{2+} fluorescence intensity at 0.1 MPa showed no acute change, while photobleaching was observed since the slope of linear regression for the normalized fluorescence intensity at 0.1 MPa was $(-5 \pm 1) \times 10^{-4}$ s^{-1}

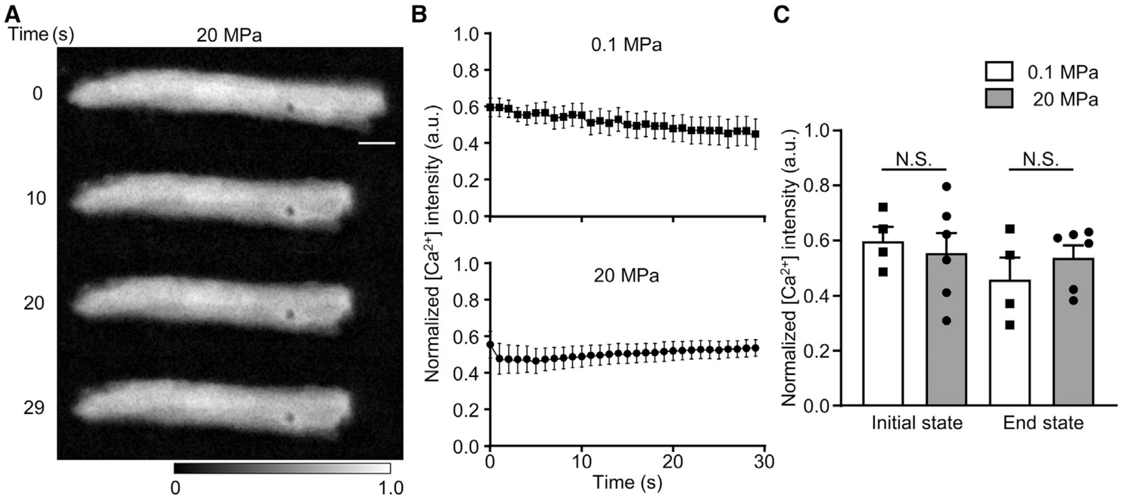

FIGURE 3 Alteration in intracellular Ca^{2+} of cardiomyocytes under high-hydrostatic pressure. (*A*) Sequential grayscale images of Ca^{2+} fluorescence intensity in cardiomyocytes at 20 MPa pressure; Ca^{2+} intensity remains constant with time. Scale bars, 10 μm. (*B*) Time-dependent change of normalized Ca^{2+} intensity to maximal Ca^{2+} intensity in cardiomyocytes under each pressure condition (0.1 MPa [$n = 4$ cells]; 20 MPa [$n = 6$ cells]). (*C*) Comparison of Ca^{2+} intensity between 0.1 and 20 MPa ($n = 4$ and 6 cells, respectively) at the initial (t = 0 s) and end (t = 30 s) states. N.S., not significant. Images were recorded at 10 frame s^{-1}. Refer to Videos S8 and S9.

(mean $\pm$ fitting error) (Fig. 3 *B* and Video S7). The fluorescence intensity at 20 MPa was not significantly different from that at 0.1 MPa (Fig. 3 *C*). These results are consistent with those of a previous study in skeletal muscle showing that cytosolic Ca^{2+} fluorescence remains constant at an applied pressure of 10–20 MPa. (44). Pressure-induced skeletal muscle contraction is observed at low Ca^{2+} levels (pCa > 6.0), while pressure-induced muscle relaxation is observed at high Ca^{2+} levels (pCa < 5.5) (55). Although we did not observe a significant increase in cytosolic Ca^{2+} at high pressure in the present study, we noted that the cytosolic Ca^{2+} intensity slightly increased at 20 MPa because the slope of linear regression for the normalized fluorescence intensity at 20 MPa was $(3 \pm 1) \times 10^{-4}$ s^{-1} (mean $\pm$ fitting error) (Fig. 3 *B*). One possibility is that there was a slow increase in leakage from Ca^{2+} stores under high pressure without an acute transient increase in $[Ca^{2+}]_i$ since mechanical stresses, such as stretch and hydrostatic pressure, have been reported to cause Ca^{2+} leaks in cardiomyocytes and *Chlamydomonas* cells (33,42). The accumulation of the Ca^{2+} leak, generated by high pressure, may cause a slow and slight increase in intracellular Ca^{2+}, binding to the tropomyosin-troponin complex to initiate actomyosin interaction. Another possibility is that the increase in fluorescence intensity is attributed to the increase in intracellular chromophore concentration with the increase in myocyte thickness when it shortens. However, the lack of a significant difference in cytosolic Ca^{2+} between 0.1 and 20 MPa due to the decrease in fluorescence intensity under high pressure would compensate for this increase. Therefore, our findings suggest that high-pressure-induced contraction can be observed in cardiac myocytes without an acute transient increase in $[Ca^{2+}]_i$ induced by CICR.

A model of pressure-induced slow contraction

The effect of high hydrostatic pressure on contraction in striated muscle has been well studied for approximately 100 years (21–26,56). Hydrostatic pressure between 5 and 40 MPa augments contraction in the striated muscle, and pressures up to 240 MPa promote subdomain unfolding of tropomyosin, indicating that high pressure might activate the tropomyosin-troponin complex and promote binding to Ca^{2+} (21–27). Meanwhile, pressures up to 200 MPa do not accelerate the myosin binding to actin, although high pressures up to 100 MPa activate myosin ATPase activity (28,29).

We propose the following model. Applied pressure causes part of the tropomyosin-troponin complex to dissociate from actin filaments (1,2). Several myosin-binding sites on the actin filament are thus exposed, which promotes actomyosin interactions (3,11,49). Since only a limited number of myosin can interact at a given time, the cardiomyocyte shortens with a slow speed under high pressure. In addition to the tropomyosin-troponin complex, the applied pressure might affect titin molecules, which interact with actin filaments, affecting the slow contraction of cardiomyocytes under high pressure [57–60]. Therefore, the underlying mechanism of the pressure-induced contraction is considered to be unique from that of the physiological contraction of the muscle. Within relaxed skeletal muscle, it has been observed that passive tension increases modestly at high pressures up to 10 MPa. These results further suggest that high pressure initiates contractions (25,61). This type of response could be conserved in the striated muscle.

In this study, we increased hydrostatic pressures up to 20 MPa, which is significantly higher than physiological

hydrostatic pressure derived from blood pressure. However, this pressure is insufficient to alter the tertiary structure and enzymatic activity of contractile proteins (62–67). Skeletal muscle myosin at 20 MPa still exhibits ATPase activity and conserves its motility functions (28). Other ATP-driven molecular motors also maintain roughly the same activity as under ambient pressure (33,68). Meanwhile, the application of such relatively low pressure can enhance protein hydration by increasing the water density (41), which weakens the intermolecular interactions between protein molecules. For example, the application of ∼20 MPa of pressure has been shown to depolymerize microtubules in vivo and in vitro (68,69), suggesting that water molecules invade into mutual binding sites between tubulin molecules. In addition, it has been reported that the application of only 1 MPa of pressure immediately induces nuclear localization of DAF-16 in *C. elegans* (35). Although the pressure used in this study is not enough to change the protein structure and function, there is no doubt that it alters intracellular processes. Our model remains speculative due to a lack of direct evidence of pressure sensitivity on contractile proteins at high pressure. Therefore, further studies are required to clarify the detailed mechanism of pressure-induced slow contraction using a combination of high-pressure microscopy and purified contractile proteins, including actin, myosin, tropomyosin, and troponin.

CONCLUSION

This study is, to our knowledge, the first to report live cardiomyocyte dynamics under high hydrostatic pressure. Our data show that high hydrostatic pressure perturbs the steady state of cardiac muscle cells. The pressure-induced slow contraction in cardiomyocytes is caused by the initiation of actomyosin interaction under high pressure without the acute transient increase in $[Ca^{2+}]_i$ induced by CICR.

SUPPORTING MATERIAL

Supporting material can be found online at https://doi.org/10.1016/j.bpj.2022.07.016.

AUTHOR CONTRIBUTIONS

Y.Y. and M.M. designed the project. M.M. constructed a high-pressure vessel system. M.N. and M.M. constructed a high-pressure microscope system. Y.Y., H.K., and M.M. conducted all experiments. Y.Y., H.K., and K.K. prepared the cardiomyocytes isolated from mouse hearts. Y.Y. prepared the draft. M.N. and M.M. edited the manuscript. Y.Y., M.N., T.K., and M.M. analyzed the results, and all the authors discussed the results. G.I., A.T., and K.N. supervised the study.

ACKNOWLEDGMENTS

The authors would like to thank Ms. Masumi Furutani (Central Research Laboratory, Okayama University Medical School, Japan) for technical assistance with the histological preparation for transmission electron microscopy and Dr. Masahiro Fujihashi for technical support. The authors also thank Dr. Armen Mekhdjian for reading our manuscript and Dr. Shinichi Ishiwata (Waseda University, Japan) and Dr. Toshiyuki Oda (University of Yamanashi, Japan) for helpful discussions. This work was funded by grants from the Japan Society for the Promotion of Science (JSPS KAKENHI Grant Numbers JP18K12033, JP21K12645, and JP21H05128 to M.M.; JP16K04908, JP19H02566, and JP22H01922 to M.N.; JP19K16485 and JP21K15338 to Y.Y.; JP21H04960 to K.N.), Innovative Science and Technology Initiative for Security Grant Number JPJ004596 to K.N., and the Akiyama Life Science Foundation (Grant No. 112-010), Asahikawa Medical University grants for anti-aging research projects (Grant No. 30-8), and Suzuken Memorial Foundation (Grant No. 21-111) to Y.Y.

DECLARATION OF INTERESTS

The authors declare no competing interests.

REFERENCES

1. Oda, T., H. Yanagisawa, and T. Wakabayashi. 2020. Cryo-EM structures of cardiac thin filaments reveal the 3D architecture of troponin. *J. Struct. Biol.* 209:107450.
2. Yamada, Y., K. Namba, and T. Fujii. 2020. Cardiac muscle thin filament structures reveal calcium regulatory mechanism. *Nat. Commun.* 11:153.
3. Kobayashi, T., L. Jin, and P. P. de Tombe. 2008. Cardiac thin filament regulation. *Pflugers Arch.* 457:37–46.
4. Gordon, A. M., E. Homsher, and M. Regnier. 2000. Regulation of contraction in striated muscle. *Physiol. Rev.* 80:853–924.
5. Gunning, P., G. O'neill, and E. Hardeman. 2008. Tropomyosin-based regulation of the actin cytoskeleton in time and space. *Physiol. Rev.* 88:1–35.
6. Tobacman, L. S. 2021. Troponin revealed: uncovering the structure of the thin filament on-off switch in striated muscle. *Biophys. J.* 120:1–9.
7. Bers, D. M. 2002. Cardiac excitation–contraction coupling. *Nature.* 415:198–205.
8. Bers, D. M., and T. R. Shannon. 2013. Calcium movements inside the sarcoplasmic reticulum of cardiac myocytes. *J. Mol. Cell. Cardiol.* 58:59–66.
9. Bers, D. M. 2014. Cardiac sarcoplasmic reticulum calcium leak: basis and roles in cardiac dysfunction. *Annu. Rev. Physiol.* 76:107–127.
10. Yamaguchi, Y., G. Iribe, ..., K. Naruse. 2017. Role of TRPC3 and TRPC6 channels in the myocardial response to stretch: linking physiology and pathophysiology. *Prog. Biophys. Mol. Biol.* 130:264–272.
11. Sequeira, V., and J. van der Velden. 2015. Historical perspective on heart function: the frank–Starling law. *Biophys. Rev.* 7:421–447.
12. Prosser, B. L., C. W. Ward, and W. J. Lederer. 2011. X-ROS signaling: rapid mechano-chemo transduction in heart. *Science.* 333:1440–1445.
13. Kobirumaki-Shimozawa, F., T. Inoue, ..., N. Fukuda. 2014. Cardiac thin filament regulation and the Frank–Starling mechanism. *J. Physiol. Sci.* 64:221–232.
14. Burnstock, G. 2017. Purinergic signaling in the cardiovascular system. *Circ. Res.* 120:207–228.
15. Beech, D. J., and A. C. Kalli. 2019. Force sensing by piezo channels in cardiovascular Health and disease. *Arterioscler. Thromb. Vasc. Biol.* 39:2228–2239.
16. Oyama, K., A. Mizuno, ..., S. Ishiwata. 2012. Microscopic heat pulses induce contraction of cardiomyocytes without calcium transients. *Biochem. Biophys. Res. Commun.* 417:607–612.
17. Mattson, D. L. 2001. Comparison of arterial blood pressure in different strains of mice. *Am. J. Hypertens.* 14:405–408.

18. Sharif-Naeini, R., J. H. A. Folgering, …, E. Honoré. 2010. Sensing pressure in the cardiovascular system: Gq-coupled mechanoreceptors and TRP channels. *J. Mol. Cell. Cardiol.* 48:83–89.

19. Fassina, L., G. Magenes, …, F. Naro. 2015. Modulation of the cardiomyocyte contraction inside a hydrostatic pressure bioreactor: in vitro verification of the Frank-Starling law. *Biomed. Res. Int.* 2015:542105.

20. Konhilas, J. P., T. C. Irving, and P. P. de Tombe. 2002. Frank-Starling law of the heart and the cellular mechanisms of length-dependent activation. *Pflugers Arch.* 445:305–310.

21. Edwards, D. J., and M. Cattell. 1928. THE STIMULATING ACTION OF HYDROSTATIC PRESSURE ON CARDIAC FUNCTION. *Am. J. Physiol.* 84:472–484.

22. Cattell, M., and D. J. Edwards. 1930. Reversal of the stimulating action of hydrostatic pressure on striated muscle. *Science.* 71:17–18.

23. Edwards, D. J., and D. E. S. Brown. 1934. The action of pressure on the form of the electromyogram of auricle muscle. *J. Cell. Comp. Physiol.* 5:1–19.

24. Hogan, P. M., and S. R. Besch. 1993. Vertebrate skeletal and cardiac muscle. *In* Advances in Comparative and Environmental Physiology, Effect of High Pressure on Biological Systems. A. G. Macdonald, ed Springer Berlin Heidelberg, Berlin, Heidelberg, pp. 125–146.

25. Ranatunga, K. W., and M. A. Geeves. 1991. Changes produced by increased hydrostatic pressure in isometric contractions of rat fast muscle. *J. Physiol.* 441:423–431.

26. Kress, K. R., O. Friedrich, …, R. H. Fink. 2001. Reversibility of high pressure effects on the contractility of skeletal muscle. *J. Muscle Res. Cell Motil.* 22:379–389.

27. Suarez, M. C., S. S. Lehrer, and J. L. Silva. 2001. Local heterogeneity in the pressure denaturation of the coiled-coil tropomyosin because of subdomain folding units. *Biochemistry.* 40:1300–1307.

28. Ikkai, T., and T. Ooi. 1971. Effects of pressure on ATPase of myosin A, heavy meromyosin, and subfragment 1. *Biochim. Biophys. Acta.* 234:190–198.

29. Iwasaki, T., and K. Yamamoto. 2002. Effect of high hydrostatic pressure on chicken myosin subfragment-1. *Int. J. Biol. Macromol.* 30:227–232.

30. Yamaguchi, Y., G. Iribe, K. Naruse…, 2018. TRPC3 participates in angiotensin II type 1 receptor-dependent stress-induced slow increase in intracellular Ca^{2+} concentration in mouse cardiomyocytes. *J. Physiol. Sci.* 68:153–164.

31. Nishiyama, M. 2017. High-pressure microscopy for tracking dynamic properties of molecular machines. *Biophys. Chem.* 231:71–78.

32. Okuno, D., M. Nishiyama, and H. Noji. 2013. Single-molecule analysis of the rotation of F1-ATPase under high hydrostatic pressure. *Biophys. J.* 105:1635–1642.

33. Yagi, T., and M. Nishiyama. 2020. High hydrostatic pressure induces vigorous flagellar beating in Chlamydomonas non-motile mutants lacking the central apparatus. *Sci. Rep.* 10:2072.

34. Okamoto, K., T. M. Watanabe, …, H. Fujita. 2021. Pressure-induced changes on the morphology and gene expression in mammalian cells. *Biol. Open.* 10:bio058544.

35. Watanabe, N., M. Morimatsu, …, A. Higashitani. 2020. Increased hydrostatic pressure induces nuclear translocation of DAF-16/FOXO in C. elegans. *Biochem. Biophys. Res. Commun.* 523:853–858.

36. Pasqualin, C., F. Gannier, …, V. Maupoil. 2016. SarcOptiM for ImageJ: high-frequency online sarcomere length computing on stimulated cardiomyocytes. *Am. J. Physiol. Cell Physiol.* 311:C277–C283.

37. Moo, E. K., and W. Herzog. 2018. Single sarcomere contraction dynamics in a whole muscle. *Sci. Rep.* 8:15235.

38. Iribe, G., H. Jin, K. Naruse…, 2010. Effects of axial stretch on sarcolemmal BKCa channels in post-hatch chick ventricular myocytes. *Exp. Physiol.* 95:699–711.

39. Fujii, S., M. Masanari-Fujii, …, Y. Sambongi. 2018. Commonly stabilized cytochromes c from deep-sea Shewanella and Pseudomonas. *Biosci. Biotechnol. Biochem.* 82:792–799.

40. Morikawa, T. J., M. Nishiyama, …, T. M. Watanabe. 2021. Glycine insertion modulates the fluorescence properties of *Aequorea victoria* green fluorescent protein and its variants in their ambient environment. *Biophys. Physicobiol.* 18:145–158.

41. Marshall, W. L., and E. U. Franck. 1981. Ion product of water substance, 0–1000 °C, 1–10, 000 bars New International Formulation and its background. *J. Phys. Chem. Ref. Data.* 10:295–304.

42. Iribe, G., C. W. Ward, …, P. Kohl. 2009. Axial stretch of rat single ventricular cardiomyocytes causes an acute and transient increase in Ca^{2+} spark rate. *Circ. Res.* 104:787–795.

43. Shiels, H. A., and E. White. 2008. The Frank–Starling mechanism in vertebrate cardiac myocytes. *J. Exp. Biol.* 211:2005–2013.

44. Friedrich, O., F. V. Wegner, …, R. H. A. Fink. 2006. "In situ" high pressure confocal Ca(2+)-fluorescence microscopy in skeletal muscle: a new method to study pressure limits in mammalian cells. *Undersea Hyperb. Med.* 33:181–195.

45. Boyett, M. R., J. E. Frampton, and M. S. Kirby. 1991. The length, width and volume of isolated rat and ferret ventricular myocytes during twitch contractions and changes in osmotic strength. *Exp. Physiol.* 76:259–270.

46. Radke, M. H., J. Peng, …, M. Gotthardt. 2007. Targeted deletion of titin N2B region leads to diastolic dysfunction and cardiac atrophy. *Proc. Natl. Acad. Sci. USA.* 104:3444–3449.

47. Rusman, H., B. Gerelt, …, A. Suzuki. 2007. Combined effects of high pressure and heat on shear value and histological characteristics of bovine skeletal muscle. *Asian-australas. J. Anim. Sci.* 20:994–1001.

48. O'Connell, T. D., M. C. Rodrigo, and P. C. Simpson. 2007. Isolation and culture of adult mouse cardiac myocytes. *In* Cardiovascular Proteomics. Humana Press, New Jersey, pp. 271–296.

49. Barua, B., D. A. Winkelmann, …, S. E. Hitchcock-DeGregori. 2012. Regulation of actin-myosin interaction by conserved periodic sites of tropomyosin. *Proc. Natl. Acad. Sci. USA.* 109:18425–18430.

50. Feher, J. 2012. The cellular basis of cardiac contractility. *In* Quantitative Human Physiology. Elsevier, pp. 547–555.

51. Herrmann, C., J. Wray, …, T. Barman. 1992. Effect of 2, 3-butanedione monoxime on myosin and myofibrillar ATPases. An example of an uncompetitive inhibitor. *Biochemistry.* 31:12227–12232.

52. McKillop, D. F., N. S. Fortune, …, M. A. Geeves. 1994. The influence of 2, 3-butanedione 2-monoxime (BDM) on the interaction between actin and myosin in solution and in skinned muscle fibres. *J. Muscle Res. Cell Motil.* 15:309–318.

53. Bond, L. M., D. A. Tumbarello, …, F. Buss. 2013. Small-molecule inhibitors of myosin proteins. *Future Med. Chem.* 5:41–52.

54. Schneidereit, D., H. Vass, …, O. Friedrich. 2016. Calcium sensitive fluorescent dyes fluo-4 and fura red under pressure: behaviour of fluorescence and buffer properties under hydrostatic pressures up to 200 MPa. *PLoS One.* 11:e0164509.

55. Fortune, N. S., M. A. Geeves, and K. W. Ranatunga. 1994. Contractile activation and force generation in skinned rabbit muscle fibres: effects of hydrostatic pressure. *J. Physiol.* 474:283–290.

56. Borjigin, G., S. Yamamoto, …, A. Suzuki. 2006. Changes in the immunogold electron-microscopic localization of calpain in bovine skeletal muscle induced by conditioning and high-pressure treatment. *Biosci. Biotechnol. Biochem.* 70:1249–1254.

57. Kellermayer, M. S. Z., and L. Grama. 2003. Stretching and visualizing titin molecules: combining structure, dynamics and mechanics. *In* Mechanics of Elastic Biomolecules. Springer Netherlands, Dordrecht, pp. 499–511.

58. Linke, W. A., and J. M. Fernandez. 2003. Cardiac titin: molecular basis of elasticity and cellular contribution to elastic and viscous stiffness components in myocardium. *In* Mechanics of Elastic Biomolecules. Springer Netherlands, Dordrecht, pp. 483–497.

59. Granzier, H., D. Labeit, …, S. Labeit. 2003. Titin as a modular spring: emerging mechanisms for elasticity control by titin in cardiac physiology and pathophysiology. *In* Mechanics of Elastic Biomolecules. Springer Netherlands, Dordrecht, pp. 457–471.

60. Yamasaki, R., M. Berri, …, H. Granzier. 2001. Titin–actin interaction in mouse myocardium: passive tension modulation and its regulation by calcium/S100A1. *Biophys. J.* 81:2297–2313.

61. Ranatunga, K. W., N. S. Fortune, and M. A. Geeves. 1990. Hydrostatic compression in glycerinated rabbit muscle fibers. *Biophys. J.* 58:1401–1410.

62. Boonyaratanakornkit, B. B., C. B. Park, and D. S. Clark. 2002. Pressure effects on intra- and intermolecular interactions within proteins. *Biochim. Biophys. Acta.* 1595:235–249.

63. Winter, R. 2019. Interrogating the structural dynamics and energetics of biomolecular systems with pressure modulation. *Annu. Rev. Biophys.* 48:441–463.

64. Roche, J., C. A. Royer, and C. Roumestand. 2017. Monitoring protein folding through high pressure NMR spectroscopy. *Prog. Nucl. Magn. Reson. Spectrosc.* 102-103:15–31.

65. Williamson, M. P., and R. Kitahara. 2019. Characterization of low-lying excited states of proteins by high-pressure NMR. *Biochim. Biophys. Acta Proteins Proteom.* 1867:350–358.

66. Akasaka, K., and H. Matsuki. 2015. High Pressure Bioscience. Springer Netherlands, Dordrecht.

67. Hata, H., M. Nishiyama, and A. Kitao. 2020. Molecular dynamics simulation of proteins under high pressure: structure, function and thermodynamics. *Biochim. Biophys. Acta Gen. Subj.* 1864:129395.

68. Nishiyama, M., Y. Kimura, …, M. Terazima. 2009. Pressure-induced changes in the structure and function of the kinesin-microtubule complex. *Biophys. J.* 96:1142–1150.

69. Salmon, E. D., and G. W. Ellis. 1975. A new miniature hydrostatic pressure chamber for microscopy: strain-free optical glass windows facilitate phase-contrast and polarized-light microscopy of living cells. Optional fixture permits simultaneous control of pressure and temperature. *J. Cell Biol.* 65:587–602.

Biophysical Journal, Volume *121*

Supplemental information

High hydrostatic pressure induces slow contraction in mouse cardiomyocytes

Yohei Yamaguchi, Masayoshi Nishiyama, Hiroaki Kai, Toshiyuki Kaneko, Keiko Kaihara, Gentaro Iribe, Akira Takai, Keiji Naruse, and Masatoshi Morimatsu

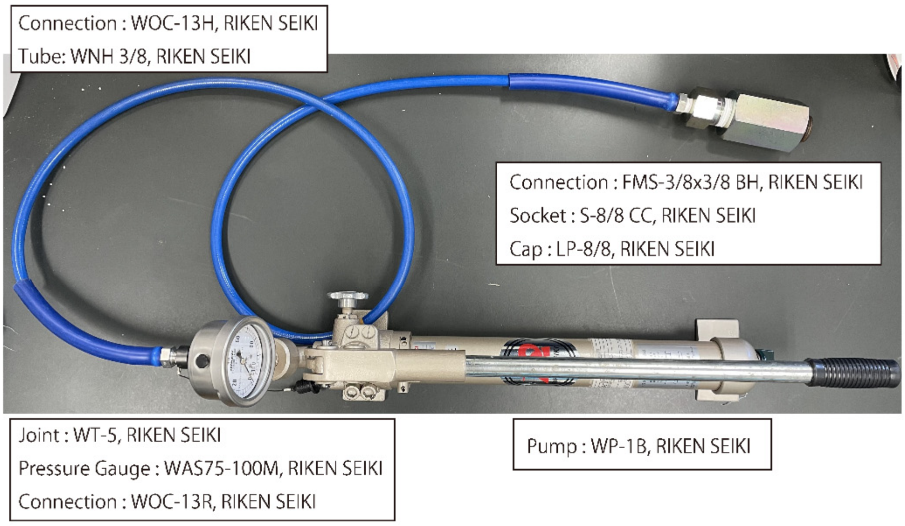

Figure S1. Image of a high-hydrostatic pressure vessel system. The high-hydrostatic pressure vessel system is composed of a pressure vessel and hand pump. The pressure vessel comprises a socket, connection, and cap (S-8/8CC, FMS-3/8×3/8 BH, and LP8/8: RIKEN SEIKI, Japan). The hand pump comprises a pump, pressure gauge, joint, and connection (WP-1B, WAS75-100M, WT-5, and WOC-13R: RIKEN SEIKI). The pressure vessel and the hand pump are connected by a tube and a connection (WNH 3/8 and WOC-13H: RIKEN SEIKI). The cells isolated from the mouse heart are placed in the pressure vessel, and the hand pump is used to apply pressure to the vessel. High hydrostatic pressure compresses the cardiomyocytes in the pressure vessel during this application, as described in the Materials and Methods section.

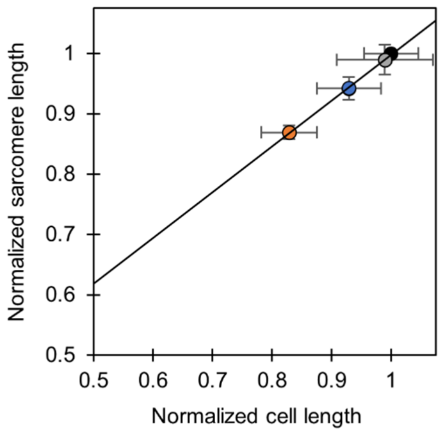

Figure S2. Correlation between cell length and sarcomere length at 0.1 (black; n = 12), 5 (gray; n = 5), 10 (blue; n = 8), and 20 MPa (orange; n = 14). Cell length is highly correlated to SL ($R^2 = 0.99$). The cell length and SL are normalized to the initial value (t = 0) at pressures. Each datapoint represents the mean and the error bars represent the SEM.

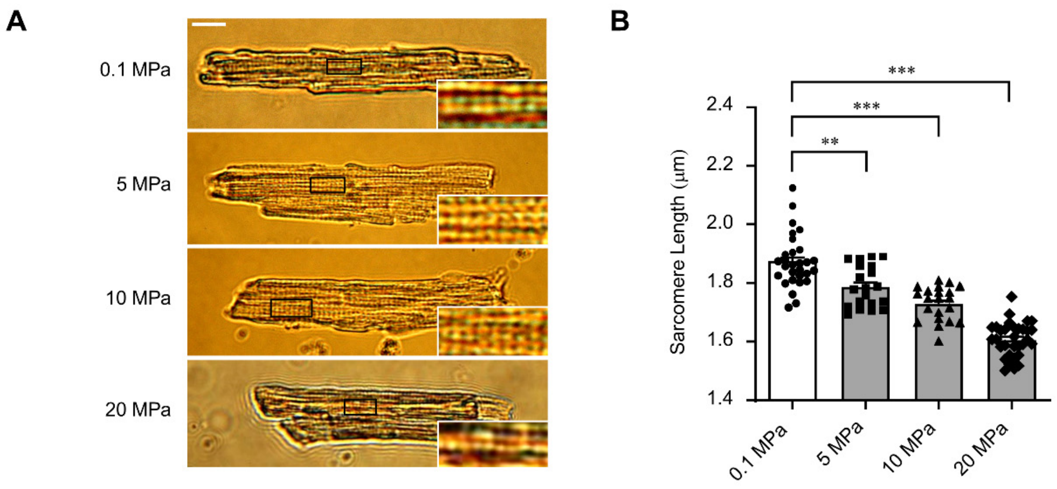

Figure S3. Morphological change in cardiomyocytes under high hydrostatic pressure using chemical fixation (A) Images of cardiomyocytes chemically fixed after exposure to atmospheric pressure (0.1 MPa) and high pressures (5, 10, and 20 MPa). The black inset is magnified in the lower right corner of the image. Scale bar: 20 µm. (B) Dose-dependent shortening of SL under high-pressure conditions (5, 10, and 20 MPa). Myocytes were

exposed to 0.1 MPa (n = 29 cells), 5 MPa (n = 20 cells), 10 MPa (n = 21 cells), and 20 MPa (n = 32 cells) pressure for 5 min. Significant shortening was observed at high-pressure conditions (5, 10, and 20 MPa). ** $p < 0.01$, *** $p < 0.001$.

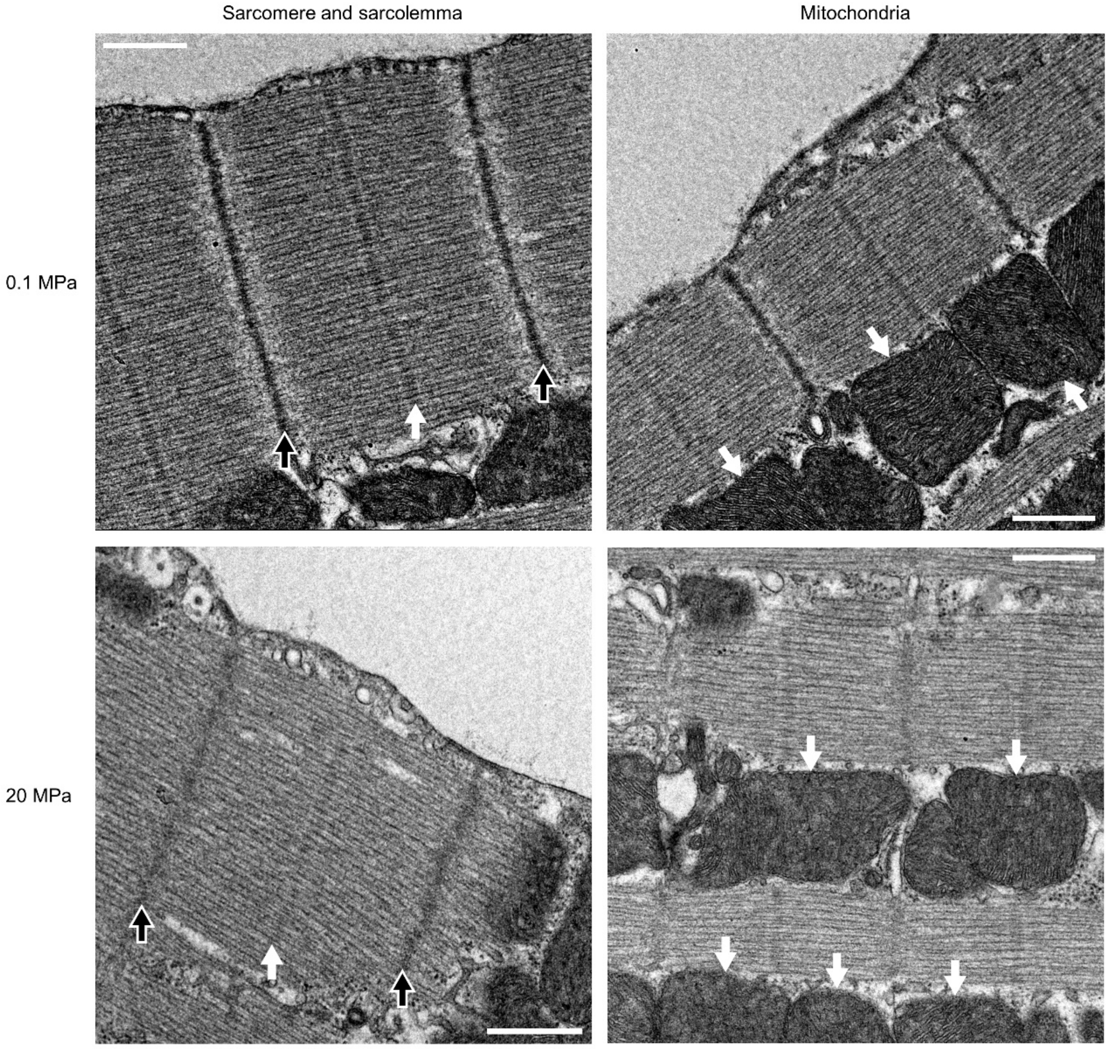

Figure S4. Transmission electron microscope (TEM) images of sarcomere, sarcolemma, and mitochondria structures in cardiomyocytes. TEM images of single cardiomyocytes were chemically fixed after exposure to atmospheric pressure (0.1 MPa) or high pressures (20 MPa). The sarcolemma and sarcomere structures, including the M line (white arrows)

and Z line (black arrows), and the inner structure of the mitochondria (white arrows) are clearly observed at atmospheric pressure (upper panels). At a pressure of 20 MPa, sarcolemma and sarcomere structures are not collapsed, while the inner structure of mitochondria (cristae) is slightly disrupted (lower panels). Scale bar: 500 nm.

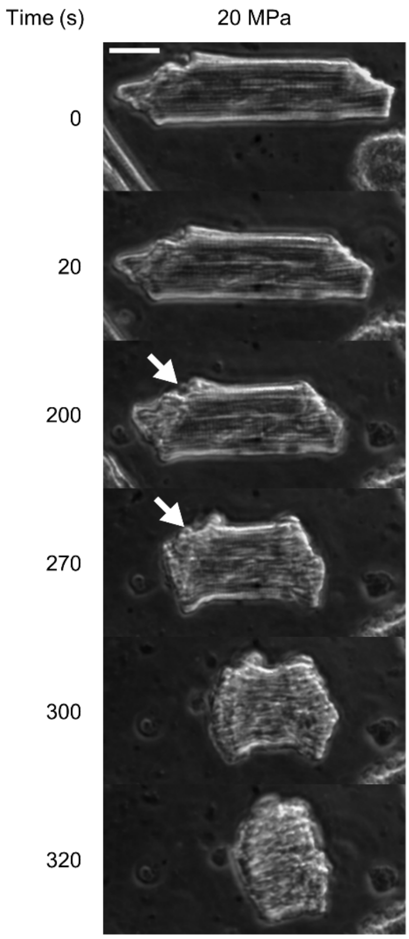

Figure S5. The collapse of cardiomyocytes under high pressure at 20 MPa. Sequential images of cardiomyocyte morphological changes under high pressure at 20 MPa show the pressure-induced cell collapse during the application from 200 to 320 s. White arrows indicate small blebbing, during which the cardiomyocytes lose their highly organized three-dimensional structure, round up, and collapse. Scale bars: 20 μm. Refer to Movie S5.

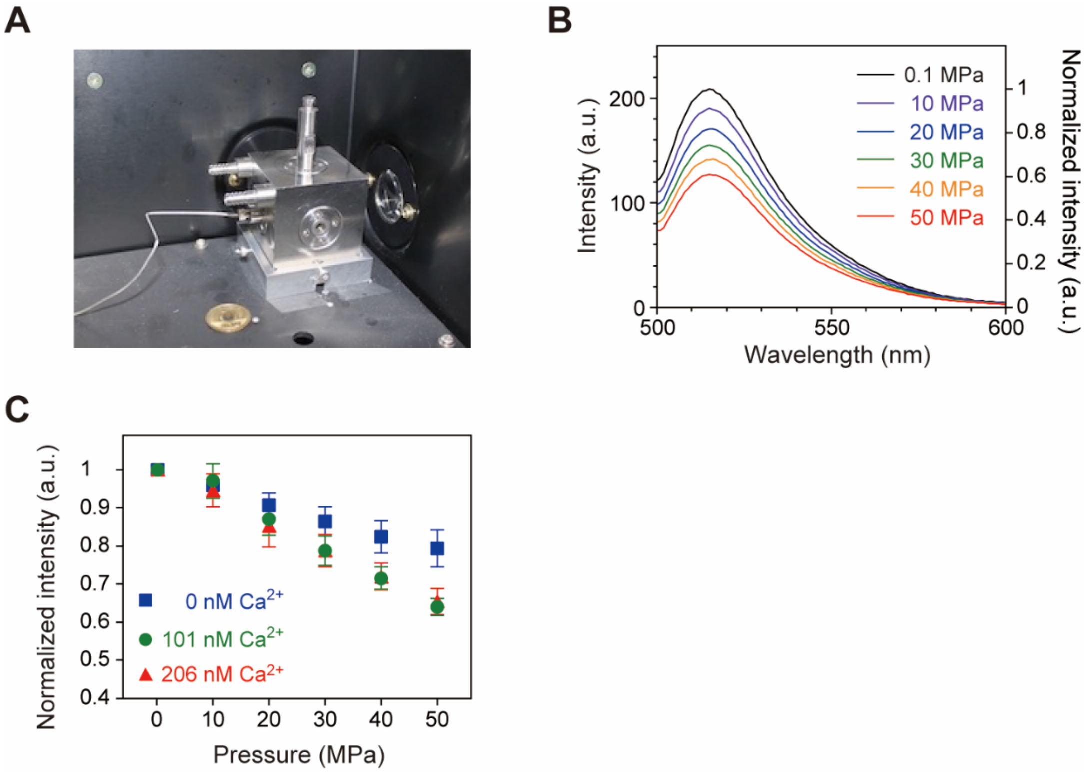

Figure S6. Effect of hydrostatic pressure on fluorescence indicator of Cal-520. (A) High-pressure chamber for spectroscopy (the diameter of the coin = 22 mm). The chamber was combined with a fluorescence spectrophotometer (F-2500, Hitachi, Japan). (B) Emission spectra of Cal-520 measured at pressures ranging from 0.1 to 50 MPa in 101 nM free Ca^{2+} concentration. The fluorescence intensities were normalized to peak values at 0.1 MPa. (C) Pressure dependence of the peak intensities at each free Ca^{2+} concentration in the buffer solution at 0.1 MPa (n = 4). Error bars represent the standard deviation.

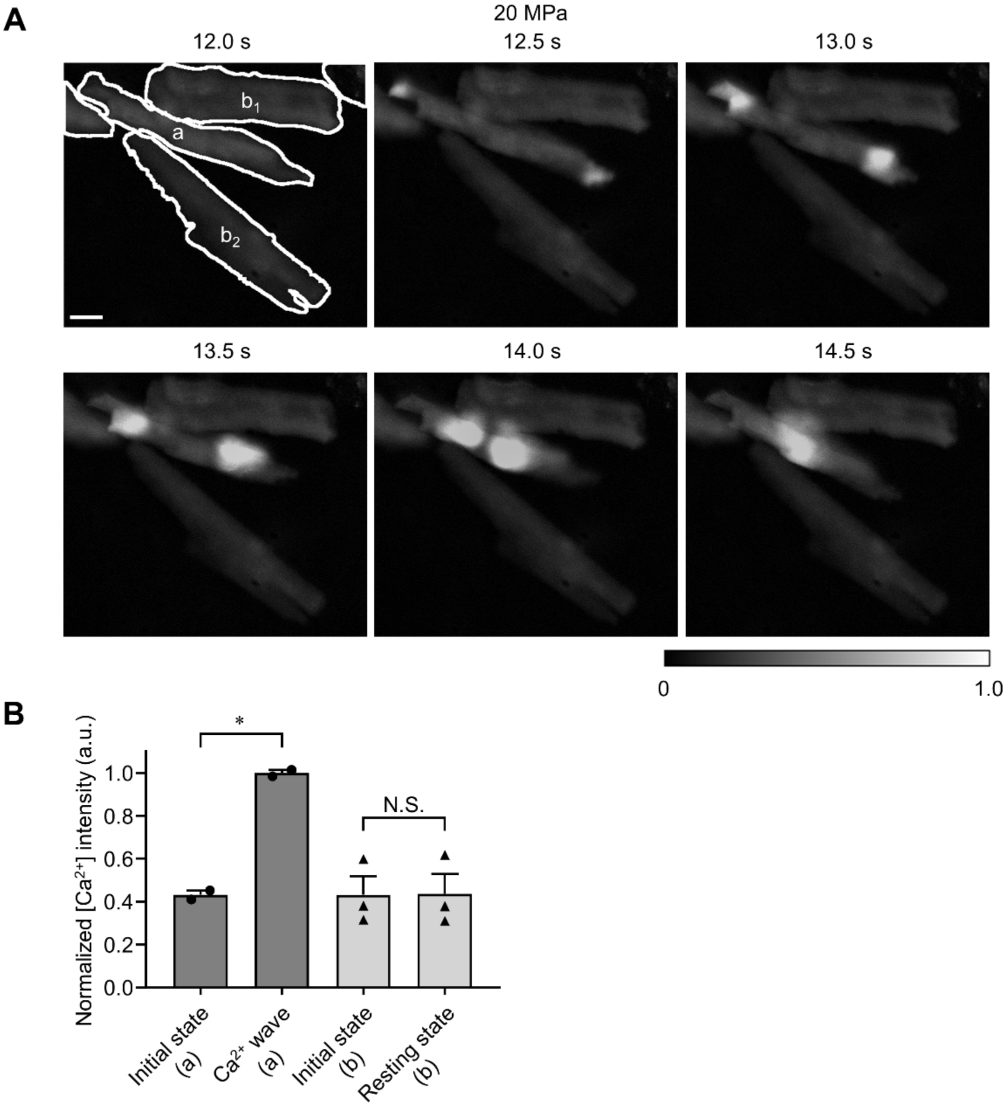

Figure S7. Qualification of detecting the acute change in cytosolic Ca^{2+} using Cal-520 AM. (A) Sequential grayscale images of cytosolic Ca^{2+} change in the cells represent Ca^{2+} wave (a) or the cells at rest (b_1 and b_2). The alteration of [Ca^{2+}] intensity is represented in grayscale. Scale bars: 20 μm. (B) In the cells with Ca^{2+} wave (a), when the Ca^{2+} wave appeared, the [Ca^{2+}] intensity was significantly elevated compared with that at an initial state. In the cells at rest (b), no significant increase in [Ca^{2+}] intensity was observed. * $p < 0.01$ vs initial state. N.S.: not significant. Refer to Movie S7.
